# Supplementation of Olive Oil and Flaxseed Oil on Blood Pressure and Inflammation in Healthy and At-Risk Adults: A Systematic Literature Review and Meta-Analysis

**DOI:** 10.2174/0115734021337760241104063418

**Published:** 2024-11-15

**Authors:** Tara B. McNabb, Ian Young, Rachel G. Newman, Roy C. Skinner, Vagner A. Benedito, Janet C. Tou

**Affiliations:** 1 School of Agriculture and Food Systems, West Virginia University, Morgantown, WV, 20506, USA;; 2 School of Occupational and Public Health, Toronto Metropolitan University, Toronto, ON, M5B 2K3, Canada;; 3 University of Chicago, Chicago, IL60637, USA;; 4 Department of Nutrition and Food Sciences, University of Vermont, Burlington, VT05405, USA

**Keywords:** Olive oil, flaxseed oil, dietary supplementation, blood pressure, hypertension, inflammation

## Abstract

**Background:**

Adding olive oil (OO) and flaxseed oil (FLO) to the diet has been reported to improve endothelial function and reduce inflammation. However, the efficacy of supplementing OO and FLO on blood pressure (BP) in normo-, pre-, and hypertensive stage 1 adults is uncertain.

**Objective:**

This study aimed to systematically review the literature on OO and FLO supplementation on BP and select inflammatory markers in healthy adults and adults at risk of hypertension.

**Methods:**

Four databases, PubMed, CINHAL, Web of Science, and Medline (Ovid), were searched from inception until October 2023 for randomized control trials (RCTs) comparing OO and FLO supplementation in normotensive or adults at risk of hypertension. The outcomes included were systolic blood pressure (SBP) and/or diastolic blood pressure (DBP) and at least one inflammatory marker, C-reactive protein (CRP), interleukin6 (IL6), or tumor necrosis factor alpha (TNFα). The risk of bias was assessed using version 2 of the Cochrane risk of bias tool for RCTs, publication bias visualization was performed using funnel plots, and meta-analysis was completed to generate average estimates of effects in 2024.

**Results:**

Seventeen RCTs, comprising 14 studies on OO and 3 on FLO, met the inclusion criteria. Meta-analysis using a random-effects model reported no significant effect on SBP n=17 mean difference (MD) -0.48; 95% CI: -1.76, 0.80; *p*=0.65, *I^2^*=0%) and DBP (n=16, MD -0.47; 95% CI: -1.33, 0.39; *p*=0.65, *I^2^*=0%) or inflammatory markers, CRP (n=8, MD 0.11; 95% CI: -1.18, 0.40; *p*=0.98, *I^2^*=0%), IL6 (n=3, MD -0.15; 95% CI: -0.57, 0.27; *p*=0.87, *I^2^*=0%), and TNFα (n=3, MD-0.08; 95% CI: -0.12, -0.03; *p*=0.98, *I^2^*=0%).

**Conclusion:**

Longer-duration, higher-dose, and larger-scale RCTs are needed to better understand the efficacy of OO and FLO supplementation on BP. Further insight will better inform dietary supplement use for preventing hypertension.

## 1 INTRODUCTION

Hypertension is the most important modifiable risk factor for preventing cardiovascular disease (CVD) and premature death worldwide [1]. The American Heart Association (AHA) 2017 guidelines define four categories of blood pressure (BP): 1) normal systolic blood pressure (SBP) is <120 mmHg and diastolic blood pressure (DBP) is <80 mmHg, 2) elevated *(i.e.*, prehypertension) is SBP 120–129 mmHg and DBP <80 mmHg, 3) stage 1 hypertension is SBP 130–139 mmHg and DBP 80–89 mmHg, and 4) stage 2 hypertension is SBP ≥ 140 mmHg and >90 mmHg. In clinical practice, patients with normal blood pressure are assumed to be at minimal risk for hypertension. However, Whelton *et al.* [2] found that the risk of hypertension increases even within the normal BP range. This study of 1,457 multi-ethnic participants who met the inclusion criteria of SBP levels of 90-129 mmHg with no CVD risk factors or use of antihypertensive medication showed a dose-effect relationship between CVD and SBP levels beginning at 90 mmHg [2]. Further, a meta-analysis consisting of 61 cohort studies and 1 million adults reported increased CVD risk starting at the normal SBP/DBP of 115/75 mmHg [3].

Individuals at risk of CVD include those who are overweight/obese, prehypertensive, maintain unhealthy diets, and/or are physically inactive. Dietary patterns, such as Dietary Approaches to Stop Hypertension (DASH) or the Mediterranean diet (MedDiet), have been shown to lower BP; however, adherence may be difficult, particularly if these diets are culturally unfamiliar [4]. Single nutrients can also have positive effects on BP. A long-standing AHA recommendation is to replace saturated fatty acids with n-3 polyunsaturated fatty acids (n-3 PUFA) and monounsaturated fatty acids (MUFA) to lower the incidence of CVD [5]. Olive oil (OO) is rich in MUFA oleic acid, while flaxseed oil (FLO) is comprised of only ~20% MUFA, mainly as oleic acid. Instead, FLO is high (>70%) in the n-3 PUFA, α-linolenic acid (ALA), which can be further metabolized to the bioactive long chain n-3 PUFAs, eicosapentaenoic acid (EPA) and docosahexaenoic acid (DHA) [6]. Both oleic acid and ALA have been reported to lower CVD risk and inflammation [7]. Complementary therapies, such as n-3 PUFA supplements, have attracted public attention for helping to prevent and manage chronic diseases safely and cost-effectively [8, 9]. According to the 2011-2014 National Health and Nutrition Examination Survey data, 52% of United States (U.S.) adults (n=11,024) reported taking at least one dietary supplement with their primary motivation being overall health and wellness [10].

Hypertension causes chronic inflammation that contributes to oxidative stress, vascular endothelial damage, and microcirculation remodeling [11]. A cross-sectional study of 196 healthy subjects investigating independent associations between different inflammatory markers and hypertension found interleukin 6 (IL6) and tumor necrosis factor-alpha (TNFα) to be independent risk factors for the development of hypertension in apparently healthy subjects [12]. Several cohort studies of normotensive individuals have found that elevated C-reactive protein (CRP) predicts the development of hypertension [13]. Studies found that participants with rheumatoid arthritis who experience a higher burden of CVD due to hypertension benefited from supplementation with a natural anti-inflammatory compound that reduced CRP, IL6, and TNFα [8]. To determine whether OO and FLO have similar anti-inflammatory actions for lowering BP, the objective of this systematic literature review (SLR) and meta-analysis was to evaluate randomized controlled trials (RCTs) examining OO and/or FLO supplementation in normotensive, prehypertensive, or untreated stage 1 hypertensive individuals. The outcomes assessed included SBP, DBP, and inflammation markers, CRP, IL6, and TNFα. Understanding the effectiveness of OO and FLO supplementation on BP can potentially decrease healthcare costs and the future risk of developing CVD.

## 2 MATERIAL AND METHODS

This study followed the preferred reporting items for systematic reviews and meta-analyses (PRISMA) guidelines [14]. The protocol for this study is provided in Appendix I. Table **1** outlines the Population, Intervention, Comparison, Outcomes, and Study design (PICOS) framework used to define the research question and the SLR inclusion and exclusion criteria.

This SLR is registered in PROSPERO registry number CRD42024588086.

### 2.1 Data Sources and Search Strategy

In consultation with a librarian, a comprehensive search strategy was developed using a combination of pre-tested search terms. Controlled vocabulary terms included medical subject heading (MESH) terms as well as free-text terms that included phrase searching, Boolean, and proximity operators developed based on the PICOS framework (Table **1**). A search was implemented in PubMed, Web of Science, CINHAL, and Medline (Ovid) from the inception of each database to October 15, 2023. The full search strategies for each database are provided in Appendix II. There was no restriction for the publication year. A hand search in relevant journals and Google Scholar was conducted. To identify ongoing and unpublished studies, the website clinicaltrials.com was searched. Studies identified through the database search were uploaded into the reference manager, Zotero (George Mason University, VA, U.S.), and duplicate studies were removed. Citations were imported into a systematic review screening software program (Covidence Systematic Review Software, Veritas Health Innovation, Melbourne, Australia) to conduct screening for eligibility and data extraction.

### 2.2 Eligibility Criteria

The population of interest was adults aged >18 years old who were normal weight or overweight/obese but otherwise healthy individuals who met the AHA categories for normo-, pre-, and stage 1 hypertension. As better compliance is expected with supplements rather than diet interventions, only studies that used capsules or vials of OO or FLO were included. Excluded were interventions that involved OO and FLO supplementation in combination with diet patterns (*e.g.*, MedDiet) that could confound the BP effects of the oils alone. OO included all oil grades (*e.g.*, EVOO, virgin, refined, and olive oil pomace) but excluded oil blends or oil enriched with other compounds that can influence BP (*e.g.*, high oleic, high phenolics) other than <2% vitamin E to prevent rancidity. Study interventions compared no or other oils provided in the identical form as the OO or FLO capsules, and vials were included. Interventions lasting less than 2 weeks were excluded. The primary outcomes were SBP and/or DPB at baseline and the end of the intervention period. Secondary outcomes were at least one inflammation marker, including CRP, IL6, or TNFα. Studies that included inflammation markers but not BP and those that did not include inflammation marker values at baseline and at the end of the intervention period were also excluded. Study designs included RCTs, parallel or crossover. Additionally, only original peer-reviewed journal articles published in English were included.

### 2.3 Screening and Study Selection

Articles obtained utilizing the search strategies were imported into Covidence Systematic Review Software. Two reviewers (TBM and JCT) independently screened the title and abstracts to identify studies that met the eligibility criteria using standardized piloted screening forms in Covidence (Appendix **III**). Studies that met the criteria were selected for full-text screening (Appendix **IV**). Any conflicts were resolved by discussion between reviewers and, if necessary, in consultation with a third reviewer, RGN.

### 2.4 Study Description and Data Extraction

Data was independently extracted by both reviewers (TBM and JCT) using the Covidence Systematic Review Software and standardized piloted extraction forms (Appendix V). For each RCT, the following data were extracted: author, year, RCT design (*i.e.*, crossover or parallel), intervention duration, oil type and grade, supplement dosage, number of participants, and participant characteristics (i.e., gender, age, whether race/ethnicity was specified) and details on outcomes of interests (*i.e.*, SBP, DBP, CRP, IL6, and TNFα). RGN was contacted to make the final decision in case of any conflict. All outcomes of interest that could potentially be used for meta-analysis were compiled in Microsoft Excel spreadsheets (Microsoft Corporation, WA, U.S.) for analysis by IY.

### 2.5 Risk of Bias Assessment

Two authors (TBM and JCT) independently assessed the risk of bias for each RCT based on the Cochrane Risk of Bias tool-version 2 (RoB 2) criteria [15]. Bias arising from randomization, intervention deviations, missing data, outcome measurement, and reported results was evaluated. Judgement of the risk of bias in each RCT was assigned to one of three levels for each domain: low risk of bias, some concerns, or high risk of bias. RGN was contacted to make the final decision in case of any conflict. The risk of bias results were added to the Cochrane RoB2 Excel template, and a traffic light plot and summary plot were generated using the Robvis visualization tool [16].

### 2.6 Data Analysis

Meta-analysis was conducted using a random-effects model with restricted maximum likelihood estimation of variance and a Hartung-Knapp (HK) adjustment to calculate raw mean differences of each outcome [17]. Given that OO was the control or the placebo group in almost all the studies, the meta-analysis was conducted on the difference between the baseline and post-treatment values in groups that received either OO or FLO. As correlations between pre- and post-measures were not reported in any study, a paired analysis was not possible, and meta-analysis was conducted on mean differences (MD), treating the measures as independent. When only standard errors or confidence intervals were reported instead of SDs in studies, SDs were estimated using the formulas in the Cochrane Handbook [18]. OO and FLO treatments were combined in the same meta-analysis to calculate an overall average effect for these treatments. MDs were considered significant if the 95% confidence interval (CI) excluded the null. The heterogeneity of effect estimates was assessed using *I*^2^, which describes the proportion of variation that cannot be explained by sampling error alone. *I^2^* values of 25%, 50%, and 75% correspond to low, moderate, and high degrees of heterogeneity, respectively [18]. Given the current evidence, prediction intervals were also calculated to indicate the range of expected effect sizes for future studies [19]. Assessment of potential small study effects (*e.g.*, publication bias) was evaluated using funnel plots as well as using quantitative tests, including Egger’s test, trim and fill method, when at least 10 studies were available for an outcome of interest. Meta-analysis was conducted using the “meta” package in R version 4.2.2 and the RStudio interface (version 2022.07.2) [20] using R Core Team (https://www.R-project.org/) and Posit (https://posit.co/).

## 3 RESULTS

### 3.1 Database Search

A total of 1,090 citations were identified by searching PubMed (n=485), CINAHL (n=135), Web of Science (n=322), and Medline (Ovid) (n=148). De-duplication of studies resulted in 855 citations for screening of the titles and abstracts for relevance by two independent reviewers. After screening, 220 citations met the criteria for full-text examination. A total of 203 studies were excluded after the full-text review. A total of 17 studies were included in the systematic literature review (SLR). Fig. (**1**) shows the PRISMA flowchart with reasons for excluding articles based on abstract and title screening and with reasons for excluding papers based on full article screening. Total studies with adequate data for conducting meta-analysis were n=17 for SBP, n=16 for DBP, n=8 for CRP, n=3 for IL6, and n=3 for TNFα.

### 3.2 Description of Included Studies

Table **2** shows the characteristics of the participants and the experimental design of 17 studies. The majority of RCTs used a parallel (88%) *versus* crossover (12%) study design. RCTs were mainly conducted at a single site (94%) in the U.S., Canada, the United Kingdom (U.K.), and Europe. Most (65%) studies included 25-100 participants/group, while only 12% included >100 participants/group. Participants at risk of hypertension based on risk factors of being overweight, obese, and/or prehypertensive made up 41%, while 47% of studies included healthy participants and 12% stage 1 hypertension participants that met the categorization of low risk defined by Flack *et al.* [21] as without CVD and age <65 years. Current AHA guidelines suggest that all patients with stage 1 hypertension should be provided with lifestyle therapy. However, those not achieving goal BP (<130/80 mmHg) within 6 months should continue lifestyle therapy with consideration given to the addition of medication [22]. Of the studies that provided information about diet patterns, 50% recommended that participants continue consuming their normal diets during OO or FLO supplement interventions. Oil grade used in capsules was reported in only 29% of studies.

Table **3** details the intervention and outcomes results. Three studies examined FLO supplementation. In all studies, FLO was the treatment group being compared to a control/placebo. In contrast, of the 14 RCTs that used OO supplementation, 11 studies used OO as the control/placebo when compared to treatment of either fish oil, EPA, or DHA. Of the RCTs, 53% had an intervention duration of ≤1 month, with most studies lasting 3 months. Of the 14 included studies examining OO supplementation on SBP, only two reported a significant reduction [23, 24]. Of the 13 studies that examined OO supplementation and DBP, two reported a significant reduction [23, 24]. All three studies examining FLO supplementation reported no significant differences in SBP and DBP [25-27]. Of the six studies providing OO and two studies providing FLO that measured circulating CRP, none reported any significant effect. In one study providing OO and two providing FLO that measured IL6 and TNFα, only Joris *et al*. [25] found that FLO supplementation significantly reduced circulating TNFα.

### 3.3 Risk of Bias Assessment

Fig. (**2**) shows the risk of bias assessment using Cochrane’s RoB2 tool. Of the 17 RCTs that met the inclusion criteria, seven (41%) had a low risk of bias [24-30]. Six (35%) had some concerns about bias [31-36], and four (24%) had a high risk of bias [23, 37-39]. All three RCTs examining FLO supplementation had a low risk of bias [25-27]. Of the four RCTs judged at high risk of bias, two studies conducted in the eighties lacked description in three of the five bias domains [23, 38]. Across all RCTs, the domain judged at the highest risk of bias was in the measurement of outcomes (~50%) due to missing information on whether outcome assessors were blinded to the treatment assignments and the absence of details about the effectiveness of the subject blinding. The next highest risk of bias (~25%) was bias arising from the randomization process due to a lack of details about the method used in allocation concealment. Overall, 25% of the 17 RCTs were judged to be at high risk of bias.

### 3.4 Meta-Analysis and Publication Bias

Meta-analysis was performed on outcomes of interest SBP, DBP, CRP, IL6, and TNFα. Fig. (**3**) shows the forest plot results for studies (n=17) reporting SBP. Supplementation with either OO or FLO had no effect on SBP mean difference (MD) -0.48 (95% CI: -1.76; 0.80, *p*=0.65) in healthy and at-risk subjects and showed low heterogeneity (*I*^2^ = 0%). The AlSaleh *et al.* [28] study reported no significant effect on SBP and had the highest sample size. Fig. (**4**) shows the forest plot results for studies (n=16) reporting DBP. Supplementation with either OO or FLO revealed no significant effect on DBP (MD -0.47; 95% CI: -1.33, 0.39, *p*=0.60) in healthy and at-risk subjects and showed low heterogeneity (*I*^2^ = 0%). The AlSaleh *et al.* [28] study reported no significant effect on DBP and had the highest sample size.

Fig. (**5**) shows the forest plot results for secondary outcomes. Studies (n=8) reporting on an inflammatory marker, CRP revealed supplementation with either OO or FLO supplementation had no significant effect on CRP (MD 0.11; 95% CI: -0.18, 0.40, *p*=0.92) and showed low heterogeneity (*I*^2^ = 0%). The Chen *et al.* [37] study had the highest sample size. Fig. (**6A**) shows the forest plot results for studies (n=3) reporting IL6 levels, and Fig. (**6B**) shows the forest plot results for studies (n=3) reporting TNFα levels. Supplementation with either OO or FLO showed no significant effect on IL6 (MD -0.15; 95% CI: -0.57, 0.27, p=87) or TNFα (MD -0.08; 95% CI: -0.12, -0.03, *p*=0.98) and showed low heterogeneity (*I*^2^ = 0%).

Fig. (**7A**) shows a funnel plot of the variability of the individual studies measuring SBP against the effect size. Most of the studies in the funnel plot are on the upper part, which displays larger studies with greater precision, and fewer on the base part, which displays studies with lower precision. Studies are roughly symmetrical left and right and cluster close to the null, indicating no effect. All studies measuring SBP lay within the 95% confidence diagonal dotted line and within the white-shaded region, indicating a significance of *p*<0.01. Fig. (**7B**) shows a funnel plot of the individual studies measuring DBP. All except one study in the funnel plot are clustered in the upper part. Studies are roughly symmetrical left and right, and studies cluster close to the null, indicating no effect. With one exception, all studies lay within the 95% confidence dotted diagonal line, indicating a significance of *p*<0.01. The Egger test showed no evidence of significant (*p*>0.5) small-study effects in the analysis of OO and FLO consumption on SBP or DBP. Funnel plots for primary outcomes, SBP, and DPB revealed no publication bias. Of the 17 studies, 15 reporting no significant effects of OO and FLO on BP were published.

**Fig. (7):**
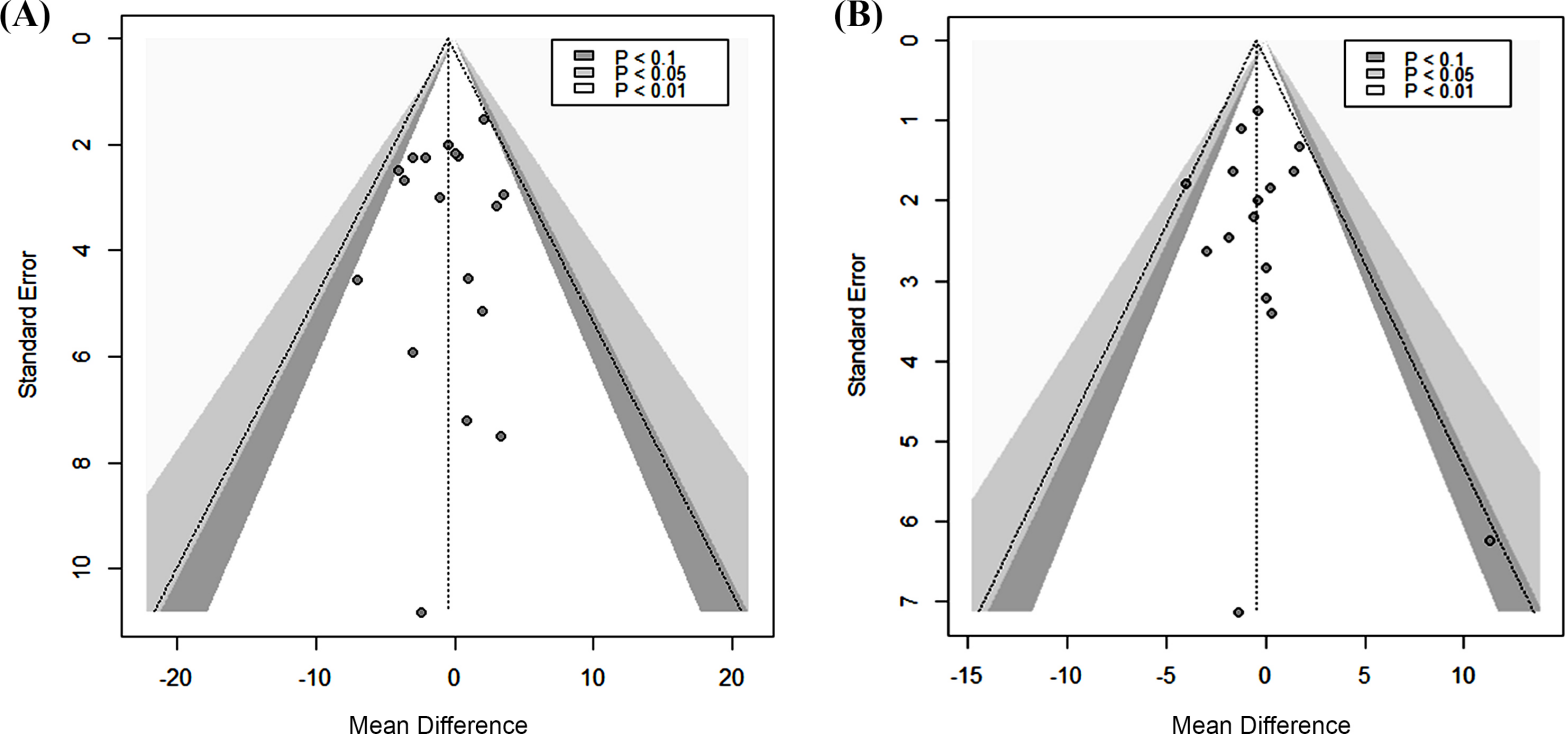
Contour-enhanced funnel plot for publication bias assessment of **A**) systolic blood pressure (SBP) and **B**) diastolic blood pressure (DBP). The Y-axis represents the standard error.

The X-axis displays the effect mean difference. The dotted center line at 0 represents no effect. The dotted diagonal lines indicate the random effects estimate and their corresponding 95% confidence intervals. The different shaded regions represent different significance levels for the effect size. The filled circles indicate individual studies (Fig. **7A**-**B**).

## 4 DISCUSSION

This SLR assessed the effect of OO and FLO supplementation on SBP, DBP, inflammatory markers, CRP, IL6, and TNFα. Of the 1,090 studies identified through database searches, 17 met the criteria for inclusion, resulting in a total of 458 participants. All RCTs, except one [31], measured both SBP and DBP. Of the 14 RCTs supplementing OO, only two studies, Brucker *et al.* [23] and Lee *et al.* [24], reported a significant SBP decrement. According to Hardy *et al.* [40], a 1 mmHg decrement in SBP can prevent cardiovascular events, and it is suggested that the management of BP should include populations below hypertension treatment thresholds. In our SLR, five studies achieving a >1 mmHg reduction in SBP reported these results as non-significant. Rogers *et al.* [35] reported OO supplementation for 6 weeks resulted in a -3.67 mmHg SBP reduction. However, the study concluded this result was nonsignificant despite the categorization of participants changing from stage 1 hypertension to prehypertension. Further, DBP was <80 mmHg at baseline and after intervention.

DBP independently influences cardiovascular events in healthy middle-aged (<65 years) participants [41]. Of our included RCTs, 65% of participants were aged <65 years, and 85% had a DBP measurement of <80 mmHg. Only two studies, Bruckner *et al.* [23] and Lee *et al.* [24], reported that OO supplementation resulted in a significant reduction in DBP -3 and -4 mmHg, respectively. A reduction of 2 mmHg in DBP in the normotensive population has been shown to lower the risk of stroke and premature CVD deaths [42]. In the current SLR, no RCT showed a >2 mmHg decrement or reported a significant effect with OO supplementation. Similarly, a previous meta-analysis of 13 RCTs comprised of mainly healthy participants reported no significant effect on DBP. However, SBP was significantly reduced when consuming EVOO compared to refined OO [43]. Of the RCTs included in our SLR, only two studies specify the grade of oil used. Chen *et al.* [37] used EVOO, while Theobald *et al*. [36] used refined OO. Refined oil has fewer bioactive compounds (*e.g.,* polyphenols) than EVOO due to the addition of chemical solvents during processing [44]. However, a meta-analysis of RCTs restricted to EVOO supplementation independent of the MedDiet revealed no significant impact on either SBP or DBP. Further, subgroup analysis based on participant health status confirmed the lack of an effect of EVOO intake on BP among hypertensive and non-hypertensive subjects [45]. In the current SLR, OO served as the placebo/control in all except three studies. OO has been extensively used as a placebo in studies investigating the effects of fish oils, a rich source of n-3 PUFAs, and a popular dietary supplement in Western countries [46].

FLO containing moderate MUFA but high in the n-3 PUFA, ALA was also investigated. None of the three RCTs investigating FLO supplementation reported significant reductions in SBP or DBP. A prior meta-analysis of five RCTs providing FLO showed no effect on DBP, but SBP was significantly lowered in participants with either stage 2 hypertension, metabolic syndrome, or hypercholesterolemia [47]. Participants included in our SLR were healthy, prehypertensive, or stage 1 hypertensive. Studies involving healthy and at-risk subjects typically demonstrate less pronounced BP reduction than individuals with underlying diseases and higher baseline BP. In support, a meta-analysis of RCTs providing ALA supplementation reported no effect on DPB but found significantly lower SBP, with more prominent effects observed in individuals with higher baseline BP [48].

Overall, the current meta-analysis of OO and FLO supplementation revealed no significant effect on SBP (MD -0.48 mmHg; 95% CI: -1.76, 0.80, *p*=0.65) and DBP (MD -0.47; 95% CI: -1.33, 0.39, *p*=0.60) in healthy and at-risk adults. Potential confounding factors were differences in protocols used to measure BP among the studies, including posture (*i.e.*, sitting or supine), the number of measurements taken, and the resting time before and between BP readings. Only two studies used 24-hour ambulatory BP [26, 34], which, by obtaining multiple readings, allows BP variability to be captured [49]. Joris *et al.* [25] examined FLO intake and measured central BP, which has been suggested to be better for predicting cardiovascular mortality [50]. In the current SLR, most studies have measured peripheral BP. Depending on the BP measurement methodology, BP results can vary by up to 20-25 mmHg [51]. Therefore, including inflammatory markers may serve as a useful adjunct measurement. An association has been reported between inflammatory markers, CRP, and prehypertension [52].

Dietary supplements are widely used by the population for disease prevention and overall health maintenance [9]. A natural substance that reduced CRP and proinflammatory, IL6 and TNFα were used as complementary therapy for managing rheumatoid arthritis, a chronic inflammatory condition associated with an increased risk of hypertension [8]. The current meta-analysis of six studies providing OO supplementation and two studies providing FLO supplementation, measuring both BP and serum CRP, found no significant effect on serum CRP level (MD 0.11; 95% CI: -0.18, 0.40, *p*=0.92). In support, a meta-analysis of RCTs providing EVOO supplementation also found no significant effect on BP and CRP [45]. Conversely, a meta-analysis of RCTs providing ALA supplements, such as flaxseed oil or FLO, reported significant reductions in SBP and CRP in participants with baseline serum CRP >3 mg/L [48]. In the current SLR, only two RCTs examining FLO supplementation met the inclusion criteria of measuring both BP and CRP. Neither of the two studies reported significant reductions in either BP or CRP [25, 27].

Inflammation markers, IL6 and TNFα, were found to be independent risk factors for hypertension in both normo- and prehypertensive subjects [52]. In our SLR, 41% of participants were prehypertensive, while 47% were normotensive. The current meta-analysis of the RCTs providing either OO supplementation found no significant effect on IL6 and TNFα levels. Similarly, a meta-analysis of RCTs providing EVOO to normotensive and hypertensive subjects also reported no significant effect on IL6 and TNFα levels [45]. In contrast, another meta-analysis reported improvement of IL6, TNFα, and CRP in healthy and unhealthy subjects; however, daily OO consumption was up to 50 mg/d and included OO supplementation combined with MedDiet [43].

In our RCTs examining FLO, Joris *et al.* [25] reported no significant effect on IL6 but a significant reduction in TNFα levels with 10g/d FLO providing ~4.7 ALA for 12 weeks. In support, a meta-analysis using flaxseed and FLO at doses of ALA > 3g/d found no effect on IL6 but a significant reduction in TNFα levels (0.45 pg/mL) [48]. However, the Joris *et al.* [25] study had fewer subjects than the Chen *et al.* [37] study reporting no significant effect on TNFα. All three RCTs examining FLO supplementation had a low risk of bias [25-27]. Overall, the risk of bias for all 17 RCTs was ~25%. Low heterogeneity in the present meta-analysis was indicated by the *I^2^* statistic of 0% for SBP, DBP, inflammatory markers, CRP, IL6, and TNFα.

Additionally, funnel plots for primary outcomes, SBP, and DPB revealed no publication bias.

### 4.1 Strengths and Limitations

The studies included in our SLR had several limitations. All RCTs in this SLR, with the exception of one, used OO as the placebo/ control, suggesting that OO doses were selected to be biologically inert. As a result, the meta-analyses were conducted only on the pre-and post-data from groups that received these treatments. However, these results could be affected by confounding variables. In RCTs, the dose of the OO supplements provided may not have been sufficient to yield significant benefits. Most studies provided 3 g/d OO supplementation, whereas MedDiet provides 1 to 4 tablespoons of OO daily or ~14 g of oil per tablespoon [53].

Guasch-Ferre *et al.* [54] reported that consuming >0.5 tablespoons or >7 g OO daily resulted in a 14% lower risk of CVD compared to non-consumers. Yet, several RCTs providing 3g/d reported lower SBP by >1 mmHg in normo-, pre-, and hypertensive stage 1 subjects. Therefore, using OO as a placebo/control in studies may not be appropriate. Additionally, reporting the oil grade used is important since processing can reduce phytochemical content. Study sample sizes were small, and only three studies investigated how FLO supplementation measured both BP and inflammation markers of interest. Furthermore, different ethnicities were only specified in 24% of studies, despite known racial differences in the susceptibility and response to interventions [55].

The strengths of our study were our criteria of including participants who were normotensive, at risk of hypertension, or classified as low-risk stage 1 hypertension. This approach reduced variability and mitigated potential sources of heterogeneity and confounding factors, such as the use of medications. Additionally, the inclusion of studies that measured inflammatory markers also required measurement of BP, while other studies focused solely on inflammatory markers. Finally, the inclusion of studies that provided OO and FLO as capsules or vials avoided the effects of cooking on oil quality and allowed better blinding and precision in dosing.

## CONCLUSION

This SLR and meta-analysis found no significant effect of OO and FLO supplementation on SBP, DBP, and inflammation markers: CRP, IL6, and TNFα in normotensive and at-risk adults. Heterogeneity among these outcomes was low, and funnel plots revealed no publication bias. However, the RCT intervention durations were short (*e.g.*, 3 months), the studies were lacking in diversity, most had some risk of bias concerns, and doses assessing OO supplementation on BP were based on OO as placebo/control doses. Further research is warranted, given that ~25–50% of adults worldwide fall into the category of prehypertensive, which increases the risk of developing hypertension threefold [56]. If efficacious, OO and FLO capsule supplementation can provide a simple intervention for lowering BP with enhanced adherence.

## AUTHORS’ CONTRIBUTIONS

It is hereby acknowledged that all authors have accepted responsibility for the manuscript's content and consented to its submission. They have meticulously reviewed all results and unanimously approved the final version of the manuscript.

## Figures and Tables

**Fig. (1):**
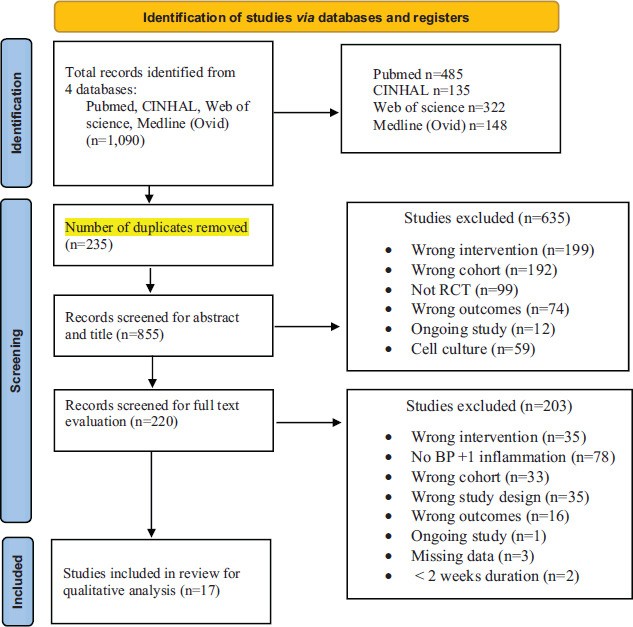
PRISMA flowchart.

**Fig. (2):**
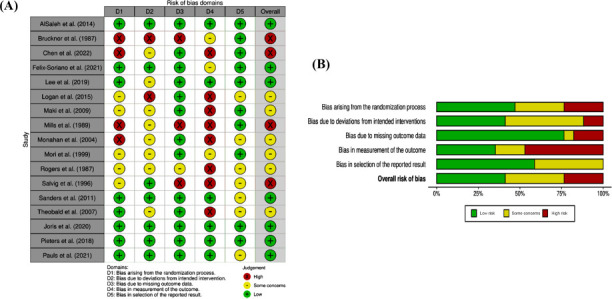
Risk of Bias **A**) Traffic Light Plot representing the risk of bias in RCTs. Individual studies are listed along the vertical axis of the plot. Different domains (D1-5) are listed along the horizontal axis. Each cell where a study intersects with a bias domain is color-coded according to the level of bias detected in that domain. Green indicates low risk, yellow signifies some concerns, and red denotes high risk. **B**) In the RoB2 summary plot, the rows of the plot correspond to different domains of bias assessment. Color coding was used to indicate the risk of bias for each domain within each study, where green represents a low risk of bias, yellow represents some concerns, and red represents a high risk of bias. At the bottom, the plot provides an overall assessment of bias across all included studies in the form of percentages indicating the number of studies with low, some concerns, or high risk of bias.

**Fig. (3):**
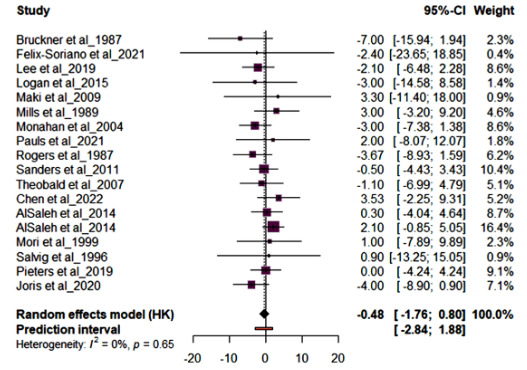
Forest plot for the pooled systolic blood pressure (SBP) from 17 randomized controlled trials (RCTs). The effect size of each study is represented by the small, solid vertical line, and its 95% confidence interval (CI) is shown by the solid horizontal line. The dashed vertical line represents the pooled prevalence, and the diamond represents its 95% CI. The size of the shaded squares symbolizes the weight each study was assigned in the pooling.

**Fig. (4):**
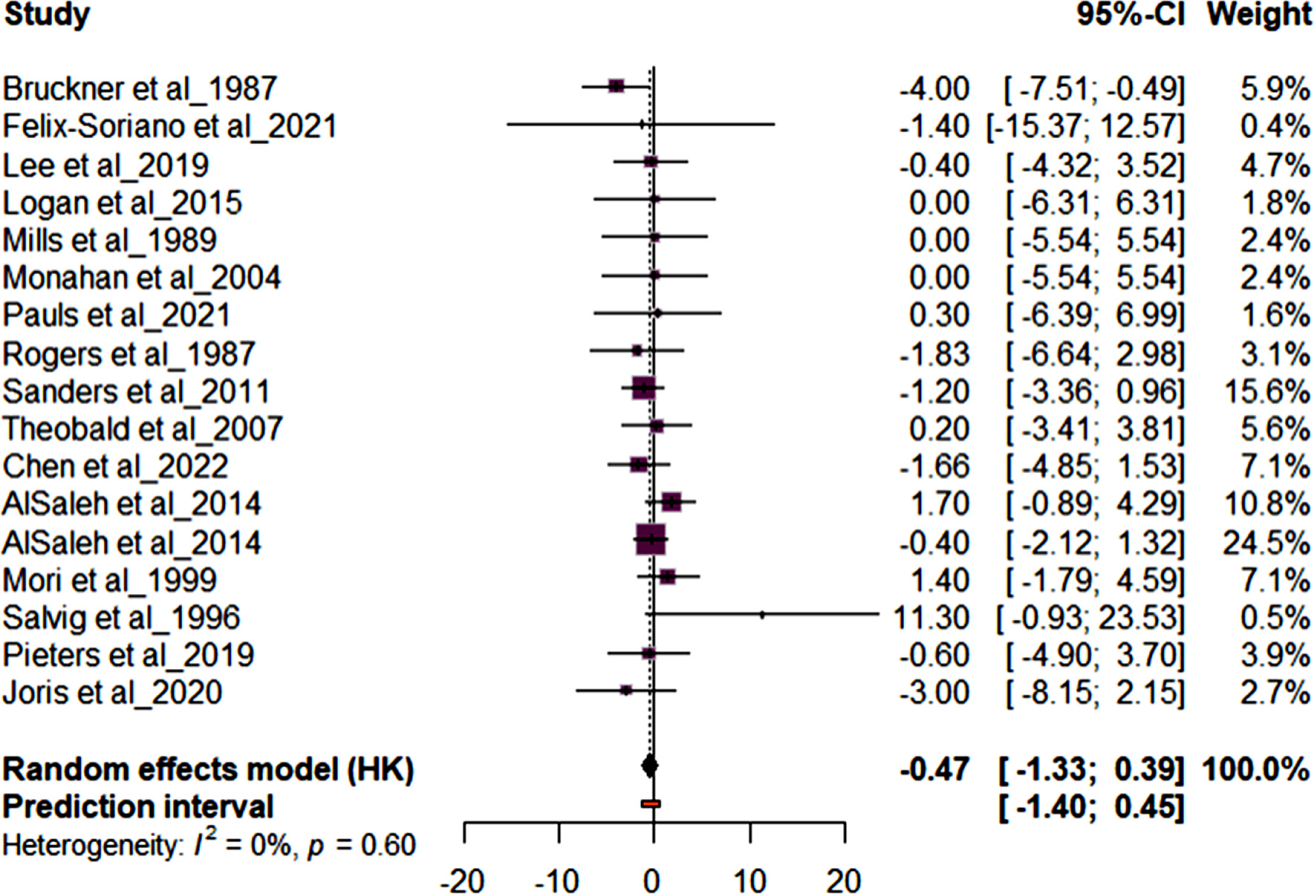
Forest plot for the pooled diastolic blood pressure (DBP) from 16 randomized controlled trials (RCTs). The effect size of each study is represented by the small, solid vertical line, and its 95% confidence interval (CI) is shown by the solid horizontal line. The dashed vertical line represents the pooled prevalence, and the diamond represents its 95% CI. The size of the shaded squares symbolizes the weight each study was assigned in the pooling.

**Fig. (5):**
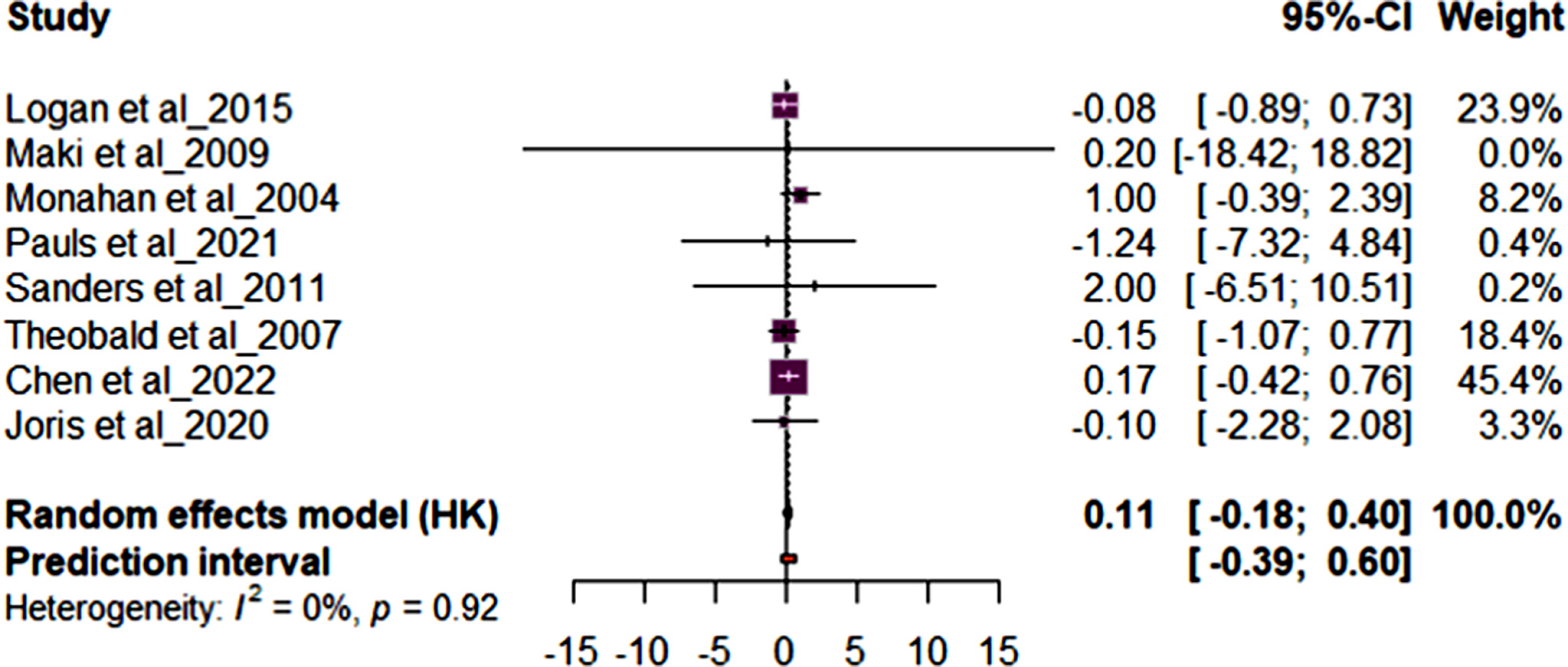
Forest plot for the pooled C-reactive protein (CRP) from 8 randomized controlled trials (RCTs). The effect size of each study is represented by the small, solid vertical line, and its 95% confidence interval (CI) is shown by the solid horizontal line. The dashed vertical line represents the pooled prevalence, and the diamond represents its 95% CI. The size of the shaded squares symbolizes the weight each study was assigned in the pooling.

**Fig. (6):**
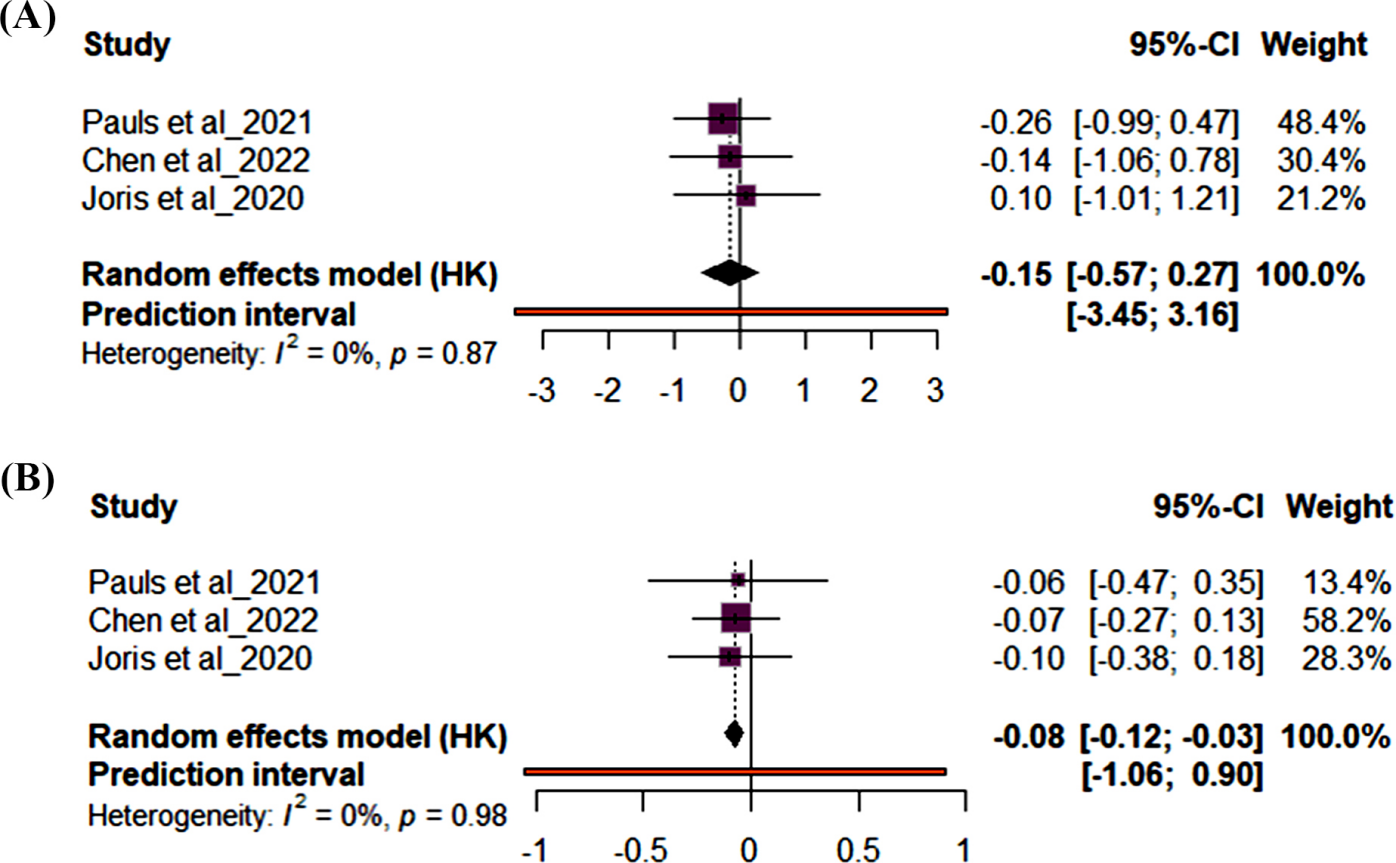
Forest plot for 3 randomized control trials (RCTs) for **A**) IL6 and **B**) TNFα. The effect size of each study is represented by the small, solid vertical line, and its 95% confidence interval (CI) is shown by the solid horizontal line. The dashed vertical line represents the pooled prevalence, and the diamond represents its 95% CI. The size of the shaded squares symbolizes the weight each study was assigned in the pooling.

**Table 1:** PICOS criteria for inclusion and exclusion of studies.

**PICOS**	**Description**
Population	adult normo-, pre-, and untreated stage 1 hypertension men and women
Intervention	olive oil and/or flaxseed oil or linseed oil
Comparison	no olive oils/flaxseed oil, different oils (fish, DHA, EPA)
Outcome	systolic and/or diastolic blood pressure, or blood pressure with at least one inflammation marker, CRP, IL6, TNFα
Study Design	randomized controlled trial, parallel or crossover

**Abbreviations:** CRP, C-reactive protein; EPA, eicosapentaenoic acid; DHA, docosahexaenoic acid; IL6, interleukin 6; tumor necrosis factor alpha (TNFα).

**Table 2:** The characteristics of the included randomized controlled trials.

**Characteristics**	**Frequency (n)**	**Percentage (%)**
**Study Design** Parallel Crossover	15 2	88% 12%
**Study Location** Single Multicenter	16 1	94% 6%
**Study Region:** United States Canada United Kingdom Europe^1^ Australia	4 4 4 4 1	23.5% 23.5% 23.5% 23.5% 6%
**Ethnicity** Specified Not Specified	4 13	24% 76%
**Number of Participants:** n= <25/group n= 25-100/group n= >100/group	4 11 2	23% 65% 12%
**Average age:** >65 years <65 years	6 11	35% 65%
**Gender** Males only Females only Both	4 4 9	23.5% 23.5% 53%
**BP Categorization:^3^** Normotensive Prehypertensive Stage 1 Hypertension	8 7 2	47% 41% 12%
**Oil Grade^4^** EVOO Virgin OO Refined OO OO pomace Refined FLO Not specified	1 0 2 0 2 12	6% 0% 12% 0% 12% 70%
**Diet Recommendations** Continue on normal diet Some recommendations No description	6 6 5	35% 35% 30%

**Note:** ^1^Spain, Demark, Netherlands; ^2^Risk include overweight, obesity, prehypertension, and/or stage 1 hypertension; ^3^Based on the American Heart Association 2017 categorization of blood pressure.; ^4^Abbreviations: EVOO, extra virgin olive oil; FLO, flaxseed oil; OO, olive oil.

**Table 3:** Characteristics of the included randomized controlled trials.

**References**	**Participants**	**Intervention Dosage**	**Intervention Duration**	**Placebo**	**Comparison Group**	**Study Results^1,2^**
AlSaleh *et al.* [26]	• Healthy adults • 45-70 yrs old • M/F n=61 • Total subjects n=254	3 g/day	1 year	OO	EPA +DHA	• NS SBP, DBP group 1&2 • SBP Before Group 1: 126.3 (95% CI: 123.6, 129) After: 126.6 (95% CI: 123.2, 130) • DBP Before Group 1: 75.2 (95% CI: 73.7, 76.6) After: 76.9 (95% CI: 74.8, 79.1) • SBP Before Group 2: 122.9 (95% CI: 121.1, 124.8) After: 125 (95% CI: 122.7, 127.3) • DBP Before Group 2: 74.5 (95% CI: 73.5, 75.5) • After: 74.1 (95% CI: 72.7, 75.5)
Bruckner *et al.* [21]	• Healthy adults • 19-40 yrs old • M (n=10) • Total subjects n=21	1.5 g oil/10 kg bwt/day	3 weeks	OO	Fish oil	• ↓ SBP, DBP (*p*<0.05) • SBP Before: 123±12 After: 116±8 • DBP Before: 82±4 After: 78±4
Chen *et al.* [35]	• Healthy adults • 18-35 yrs old • M/F (n=16) • Total subject n=43	3 g/day	1 month	No supplement	OO Fish oil	• NS SBP, DBP, IL6, TNFα • SBP Before: 115.25±6.7 After: 118.78±9.72 • DBP Before: 65.13±4.76 After: 63.47±4.44 • CRP Before: 0.463±0.475 After: 0.635±1.104 • IL6 Before: 1.11±1.47 After: 0.97±1.18 • TNFα Before: 0.82± 0.31 After: 0.75±0.26
Felix-Soriano *et al.* [27]	• At risk adults overwt/obese • 55-70 yrs old • F (n=20) • Total subjects n=71	3 g/day	4 months	OO	Fish oil -/+exercise	• NS SBP, DBP • SBP Before: 121.83 ± 19.68 Change: -2.4±10.84 • DBP Before: 80.04 ± 12.38 Change: -1.4±7.13
Lee *et al.* [22]	• Healthy adults • 18-30 yrs old • M/F (n=30) • Total subjects n=86	3 g/day	3 months	OO	EPA DHA	• ↓SBP, DBP (*p*<0.05) • SBP Before: 105.5± 8.5 After: 103.4±8.8 • DBP Before: 65.2±8 After: 64.8±7.5
Logan *et al.* [30]	• Healthy adults • 60-76 yrs old • F (n=12) • Total subjects n=24	3 g/day	3 months	OO	Fish oil	• NS SBP, DBP, CRP • SBP Before: 119±3.3 After: 116±4.9 • DBP Before: 72±1.9 After: 72±2.6 • CRP Before: 1.75±0.33 After: 1.67±0.25
Maki *et al.* [29]	• Healthy adults • 35-64 yrs old • M/F (n=25) • Total subject n=76	2 g/day	1 month	OO	Fish oil Krill oil	• NS SBP, CRP • SBP Before: 119.6±2.3 Change: 3.3±1.5 • CRP Before: 4.9±3.8 Change: 0.2±1.9
Mills *et al.* [36]	• Healthy adult subjects • 22-34 yrs old • M (n=10) • Total subjects n=30	9 capsules /day	1 month	OO	Fish oil Borage oil	• NS SBP, DBP • SBP Before: 115±1 After: 118±3 • DBP Before: 76±2 After: 76±2
Monahan *et al.* [31]	• Healthy adults • 18-35 yrs old • M/F (n=9) • Total subjects n=18	10 g/day	1 month	OO	Fish oil	• NS SBP, DBP, CRP • SBP Before: 112±2 After: 109±1 • DBP Before: 64±2 After: 64±2 • CRP Before: 8±0.5 After: 9±0.5
Mori *et al.* [32]	• At risk overweight mildly hyperlipidemic • 20-65 yrs old • M (n=20) • Total subjects n=56	4g/d	6 weeks	OO	EPA DHA	• NS SBP, DBP, IL6 • SBP Low FLO Before: 128 ±3 Change: -2±3 • SBP High FLO Before: 124±3 Change: 2±3 • DBP Low FLO Before: 76±2 Change: 2±2 • DBP High FLO Before: 73±2 Change: 2±1 • IL6 Low FLO Before: 1.64±0.21 Change: 0.44±0.08 • IL6 High FLO Before: 1.35±0.15 Change: -0.04±0.15
Rogers *et al.* [33]	• Healthy adults • 22-65 yrs old • M (n=30) • Total subject n=60	4 capsules 4x/day first week 3 capsules 3x/day remainder of trial (10-16 ml)	3-6 weeks	OO	Fish oil	• NS SBP, DBP. • SBP Before: 132.47±11 After: 128.8 ± 9.75 • DBP Before: 74.6±7.72 After: 72.77 ± 11
Salvig *et al.* [37]	• Healthy adults • 18-44 yrs old • F (n=27) • Total subjects n=81	1g/day	12 weeks	No supplement	OO Fish oil	• NS SBP, DBP • SBP Before: 125.9±1.3 After: 126.8 ±7.1 • DBP Before: 69.5±0.7 After: 80.8 ±6.2
Sanders *et al.* [28]	• Healthy adults • 45-70 yrs old • M/F (n=71) • Total subjects n=310	3 g/day	1 year	OO	EPA+DHA	• NS SBP, DBP, CRP • SBP Before: 122.6 (95% CI: 120, 125.2) After: 122.1 (95% CI: 119.2, 125.1) • DBP Before: 74.1 (95% CI: 72.6, 75.7) After: 72.9 (95% CI: 71.4, 74.4) • CRP Before: 8 (95% CI: 3,14) After: 10 (95% CI: 4,17)
Theobald *et al.* [34]	• Healthy adults • 40-65 yrs old • M/F (n=38) • Total subjects n=38	1.5 g/day	3 months	OO	DHA	• NS SBP, DBP, CRP, IL6 • SBP Before: 117.8 ± 12 After: 116.7±14.1 • DBP Before: 72.4±6.9 After:72.6±9 • CRP Before: 0.98±2.02 After: 0.83±2.09 • IL6 Before: 1760±3190 After: 1970±1770
Joris *et al.* [23]	• At risk overweight/obese • 52-68 yrs old • M/F (n=29) • Total subject n=59	10g/day	12 weeks	High oleic SO	FLO	• NS SBP, DBP, CRP ↓TNFα (*p*<0.05) • SBP Before: 129±10 After: 125±9 Change: -3 (95% CI – 8, 2) • DBP Before: 93±10 After: 90±10 Change: -3 (95% CI: 7,1) • CRP Before: 3±5.2 After: 2.9±3.0 Change: 0.15 (95% CI: -0.98,1.29) • IL6 Before: 1.2±2.1 After: 1.3±2.2 Change: 0.01 (95% CI -0.15, 0.17) • TNFα Before: 2.4±0.6 After: 2.3±0.5 Change: -0.14 (95% CI: -0.27, -0.01)
Pieters *et al.* [24]	• At risk overweight/obese • 52-68 yrs old • M/F (n=29) • Total subjects n=59	10g/day	12 weeks	High oleic SO	FLO	• NS SBP, DBP • SBP Before: 133.9±7.4 After: 133.9±9.0 • DBP Before: 85.9±8.2 After: 85.3±8.5
Pauls *et al.* [25]	• At risk adults obese • age 20-51 yrs old • F (n=21) • Total subjects n=21	4 g/day	1 month	None	FLO Fish oil	• NS SBP, DBP, CRP, TNFα, IL6. • SBP Before: 127±16.6 After: 129±16.7 • DBP Before: 80.5±11.3 After: 80.8±10.8 • CRP Before: 9.28±11.92 After: 8.04±7.74 • IL6 Before: 1.31±1.33 After: 1.05±1.08 • TNFα Before:2.01±0.74 After: 1.95±0.62

**Abbreviations:** ^1^CRP, C-reactive protein; DBP, diastolic blood pressure; IL6, interleukin-6; NS, not significantly different; SBP, systolic blood pressure; TNFα, tumor necrosis factor alpha; ^2^Blood pressure reported in mmHg; CRP reported in mg/L; IL6 and TNFα reported in pg/mL.

## Data Availability

All data generated or analyzed during this study are included in this published article.

## LIST OF ABBREVIATIONS

ALA: Alpha-Linolenic Acid
AHA: American Heart Association
BP: Blood Pressure
CVD: Cardiovascular Disease
CI: Confidence Interval
CRP: C-reactive Protein
DBP: Diastolic Blood Pressure
DHA: Docosahexaenoic Acid
EPA: Eicosahexaenoic Acid
EVOO: Extra Virgin Olive Oil
FLO: Flaxseed Oil
IL6: Interleukin 6
MD: Mean Difference
SLR: Systematic Literature Review
OO: Olive Oil
SBP: Systolic Blood Pressure
MedDiet: Mediterranean Diet
TNFα: Tumor Necrosis Factor Alpha
RCTs: Randomized Controlled Trials
RoB: Risk of Bias
MUFA: Monounsaturated Fat

## STANDARDS OF REPORTING

PRISMA guidelines were followed.

## SUPPLEMENTARY MATERIAL

PRISMA checklist is available as supplementary material on the publisher’s website along with the published article. Supplementary material is available on the publisher’s website along with the published article.
