# Supplementary material for: Supplementation of Olive Oil and Flaxseed Oil on Blood Pressure and Inflammation in Healthy and At-Risk Adults: A Systematic Literature Review and Meta-Analysis
Source: Curr Hypertens Rev. 2024 Nov 15;20(3):141–55. doi: 10.2174/0115734021337760241104063418 (PMC12079313; doi:10.2174/0115734021337760241104063418)
Supplement: Supplementary file 1 — PRISMA checklist is available as supplementary material on the publisher’s website along with the published article. Supplementary material is available on the publisher’s website along with the published article. [file CHYR-20-3-141_SD1.pdf]

Supplementary Material

Supplementation of Olive Oil and Flaxseed Oil on Blood Pressure and Inflammation in Healthy and At-Risk Adults: A Systematic Literature Review and Meta-Analysis

Tara B. McNabb[1], Ian Young[2], Rachel G. Newman[3], R. Chris Skinner[4], Vagner A. Benedito[1] and Janet C. Tou[1,*]

[1]School of Animal and Food Systems, West Virginia University, Morgantown, WV 20506, USA; [2]School of Occupational and Public Health, Toronto Metropolitan University, Toronto, ON, M5B 2K3, Canada; [3]University of Chicago, Chicago, IL 60637, USA; 4Department of Nutrition, University of Vermont, Burlington, VT 05405, USA

APPENDIX I.

Systematic literature review protocol

Background

Hypertension is the most important modifiable risk factor for preventing cardiovascular disease (CVD) and premature death worldwide (GBD Risk Factor Collaborators, 2018). In clinical practice, patients with normal blood pressure may be assumed to be at minimal risk for hypertension; however, Taler et al. (2023) found that the risk of hypertension increases even within the normal range of blood pressure measures. A meta-analysis consisting of 61 cohort studies totaling 1 million adults reported increased CVD risk starting at normal systolic blood pressure/diastolic blood pressure (SBP/DBP) (Lewington et al., 2002).

Dietary patterns have been shown to lower BP; however, adherence may be difficult, particularly if these diets are culturally unfamiliar (Ndanuko et al., 2016). Single nutrients can also have positive blood pressure effects. A long-standing recommendation of the American Heart Association is to replace saturated fats with n-3 polyunsaturated fatty acids (n-3 PUFA) and monounsaturated fatty acids (MUFA) to lower the incidence of CVD (Sacks et al., 2017). Olive oil (OO) is rich in the MUFA, oleic acid while flaxseed oil (FLO) is comprised of ~20% MUFA, mainly as oleic acid and >70% n-3 PUFA as α-linolenic acid (ALA) (Al-Madhagy et al., 2023). Both oleic acid and ALA have been reported to lower CVD risk and inflammation (Shramko et al., 2020).

A cross-sectional study of 196 healthy subjects investigating independent associations between different inflammatory markers and hypertension found tumor necrosis factor alpha (TNFα) and interleukin-6 (IL-6) to be independent risk factors for the development of hypertension in apparently healthy subjects (Bautista et al., 2005). Further, several cohort studies found elevated C-reactive protein (CRP) predicts the development of hypertension in normotensive individuals (Hage, 2014). Therefore, the objective of this systematic literature review and meta-analysis was to evaluate randomized control trials (RCTs) investigating OO and/or FLO supplementation in normotensive, prehypertensive, or low-risk (no CVD risks, no medication use) stage 1 hypertensive individuals on SBP, DBP, and inflammation markers, CRP, IL-6, and TNFα.

Significance

Understanding the impact of OO and FLO supplementation on blood pressure can potentially decrease health care costs and future risk of developing CVD.

Methodology

Review Approach, Questions, and Eligibility Criteria

This research will be conducted using standard systematic review methodology, which uses structured, transparent, and robust procedures to identify, assess, and synthesize all available evidence on a topic (Higgins & Thompson, 2002). The review question is: "what is the efficacy of OO and FLO on blood pressure and inflammation?" The PICOS (Participants, Intervention/exposure, Comparison, Outcomes, Study design) criteria was used to define the research question.

PICOS criteria for inclusion and exclusion of studies:

PICOS	Description
Population	adult normo-, pre-, and untreated stage 1 hypertension men and women
Intervention	olive oil and/or flaxseed oil
Comparison	no olive oils/flaxseed oil, different oils (fish, DHA, EPA)

Outcome	systolic and/or diastolic blood pressure, or blood pressure with at least inflammation marker, CRP, IL6, TNFα
Study Design	randomized control trial, parallel or crossover

Abbreviations: CRP, C-reactive protein; EPA, eicosapentaenoic acid; DHA, docosahexaenoic acid; IL-6, interleukin-6; tumor necrosis factor alpha (TNFα).

Inclusion Criteria

The following criteria will be used for the inclusion of original articles in the systematic review: RCT design; investigation of the impact of OO and FLO (capsule, vial) supplementation in normo-, pre-, and low-risk type I hypertensive adults. Outcomes of SBP and/or DBP and blood pressure with at least one inflammatory marker (i.e., CRP, IL-6, or TNFα) measurement at baseline and at the end of the intervention or net change during the study.

Exclusion Criteria

The following are exclusion criteria will be used for the exclusion of original articles in the systematic review: uncontrolled trials; does not measure at least one of the listed outcomes of SBP and/or DBP with at least at least one inflammatory marker (i.e., CRP, IL-6, or TNFα) measurement at baseline and at the end of the intervention or net change during the study.

Search Strategy

A comprehensive search strategy will be developed using a combination of pre-tested search terms implemented in the following bibliographic databases: PubMed, Web of Science, CINHAL, and Medline (Ovid).

**Population**	**Intervention**	**Outcome**	**Study Design**
Adults<br>Normotension<br>Prehypertension<br>Stage 1 Hypertension<br>[*synonyms for blood pressure, hypertension, systolic, diastolic, arterial*]	Dietary Supplements*<br>Flaxseed oil<br>Linseed oil<br>Alpha-linolenic acid<br>Virgin olive oil<br>Extra virgin olive oil<br>Refined oil<br>Pomace<br>Oleic acid, Monounsaturated fatty acids	Systolic blood pressure<br>Diastolic blood pressure<br>C-reactive protein<br>Interleukin-6<br>Tumor necrosis factor alpha<br>Inflammation Biomarkers	Randomized control trial<br>Randomized studies<br>Controlled trial<br>Clinical studies<br>Randomized clinical trial<br>Crossover<br>Parallel

Boolean terms of 'OR' will be used within concepts, while 'AND' will be used to connect the concepts.

The literature will be searched from inception to October 2023. A search verification strategy will also be used to ensure that no relevant articles are missed, which will include hand-searching of references of relevant articles and literature reviews on the topic. To identify ongoing and unpublished studies, the website clinicaltrials.gov was searched.

Relevance Screening and Confirmation

The titles and abstracts of citations identified during the search will be assessed for relevance using a structured screening form (Level 1 Screening). Full articles of relevant references will be confirmed for relevance and key characteristics will be extracted using another structured screening form (Level 2 Screening). This form will also pull study characteristics such as: study design, details on participant characteristics, type of supplemental intervention, and outcomes investigated.

Risk of Bias Assessment and Data Extraction

Relevant studies with extractable outcome data that meet eligibility criteria will undergo a risk of bias assessment. RCTs will be assessed using the Cochrane Collaboration's RoB2 tool (Sterne et al., 2019). Detailed quantitative data on key outcomes, factors associated with key outcomes, and the effectiveness of any supplementation interventions will be extracted using a data extraction form.

Review Management

To ensure rigor in the review process, all steps will be conducted using pre-tested tools by two independent reviewers. All references identified in the review will be de-duplicated in the reference management program (Zotero). Relevant screening, confirmation, and data extraction will be conducted using Covidence.

Meta-Analysis

Meta-analysis estimates will be calculated for any outcome that is reported in three or more studies. Meta-analysis was conducted using a random-effects model with restricted maximum likelihood estimation of variance, to calculate raw mean differences of each outcome [15]. When only standard errors or confidence intervals were reported instead of SDs in studies,

SDs were estimated using the formulas in the Cochrane Handbook. Studies reporting only medians and interquartile ranges will be excluded from analysis. Mean differences will be considered significant if the 95% confidence interval (CI) excludes the null. Heterogeneity of effect estimates will be assessed using I^2, which describes the proportion of variation that cannot be explained by sampling error alone.

REFERENCES

[1] Al-Madhagy, S., Ashmawy, N. S., Mamdouh, A., Eldahshan, O. A., & Farag, M. A. (2023). A comprehensive review of the health benefits of flaxseed oil in relation to its chemical composition and comparison with other omega-3-rich oils. *European Journal of Medical Research*, *28*(1), 240. https://doi.org/10.1186/s40001-023-01203-6

[2] Bautista, L. E., Vera, L. M., Arenas, I. A., & Gamarra, G. (2005). Independent association between inflammatory markers (C-reactive protein, interleukin-6, and TNF-alpha) and essential hypertension. *Journal of Human Hypertension*, *19*(2), 149–154. https://doi.org/10.1038/sj.jhh.1001785

[3] GBD 2017 Risk Factor Collaborators. (2018). Global, regional, and national comparative risk assessment of 84 behavioural, environmental and occupational, and metabolic risks or clusters of risks for 195 countries and territories, 1990-2017: A systematic analysis for the Global Burden of Disease Study 2017. *Lancet (London, England)*, *392*(10159), 1923–1994. https://doi.org/10.1016/S0140-6736(18)32225-6

[4] Hage, F. G. (2014). C-reactive protein and hypertension. *Journal of Human Hypertension*, *28*(7), 410–415. https://doi.org/10.1038/jhh.2013.111

[5] Higgins, J. P. T., & Thompson, S. G. (2002). Quantifying heterogeneity in a meta-analysis. *Statistics in Medicine*, *21*(11), 1539–1558. https://doi.org/10.1002/sim.1186

[6] Lewington, S., Clarke, R., Qizilbash, N., Peto, R., Collins, R., & Prospective Studies Collaboration. (2002). Age-specific relevance of usual blood pressure to vascular mortality: A meta-analysis of individual data for one million adults in 61 prospective studies. *Lancet (London, England)*, *360*(9349), 1903–1913. https://doi.org/10.1016/s0140-6736(02)11911-8

[7] Ndanuko, R. N., Tapsell, L. C., Charlton, K. E., Neale, E. P., & Batterham, M. J. (2016). Dietary Patterns and Blood Pressure in Adults: A Systematic Review and Meta-Analysis of Randomized Controlled Trials. *Advances in Nutrition (Bethesda, Md.)*, *7*(1), 76–89. https://doi.org/10.3945/an.115.009753

[8] Sacks, F. M., Lichtenstein, A. H., Wu, J. H. Y., Appel, L. J., Creager, M. A., Kris-Etherton, P. M., Miller, M., Rimm, E. B., Rudel, L. L., Robinson, J. G., Stone, N. J., Van Horn, L. V., & American Heart Association. (2017). Dietary Fats and Cardiovascular Disease: A Presidential Advisory From the American Heart Association. *Circulation*, *136*(3), e1–e23. https://doi.org/10.1161/CIR.0000000000000510

[9] Shramko, V. S., Polonskaya, Y. V., Kashtanova, E. V., Stakhneva, E. M., & Ragino, Y. I. (2020). The Short Overview on the Relevance of Fatty Acids for Human Cardiovascular Disorders. *Biomolecules*, *10*(8), 1127. https://doi.org/10.3390/biom10081127

[10] Sterne, J. A. C., Savović, J., Page, M. J., Elbers, R. G., Blencowe, N. S., Boutron, I., Cates, C. J., Cheng, H.-Y., Corbett, M. S., Eldridge, S. M., Emberson, J. R., Hernán, M. A., Hopewell, S., Hróbjartsson, A., Junqueira, D. R., Jüni, P., Kirkham, J. J., Lasserson, T., Li, T., … Higgins, J. P. T. (2019). RoB 2: A revised tool for assessing risk of bias in randomised trials. *BMJ (Clinical Research Ed.)*, *366*, l4898. https://doi.org/10.1136/bmj.l4898

[11] Taler, S. J. (2023). Lowering the Norms for Blood Pressure. *Mayo Clinic Proceedings*, *98*(3), 363–365. https://doi.org/10.1016/j.mayocp.2023.01.014

APPENDIX II. SEARCH STRATEGIES BY DATABASE.

**Database:**	**PubMed**		
**Date of Search**	**10/15/23**		
**Topic**	**Search words**	**Filters Applied**	**#Of Articles**
Olive oil/ Flaxseed oil (I)	"olive oil" [tiab] or "extra virgin olive oil" [tiab] or "virgin olive oil" [tiab] or "olive pomace oil" [tiab] or "refined olive oil" [tiab] or "pure olive oil" [tiab] or "oleic acid" [tiab] or "unrefined olive oil" [tiab] or "olive fruit oil" [tiab] or "olive extract" [tiab] or "light olive oil" [tiab] or "monounsaturated fatty acids" [tiab] or "olea europaea oil" [tiab] or "flaxseed oil" [tiab] or "flax oil" [tiab] or "linseed oil" [tiab] or "flax extract" [tiab] or "linseed extract" [tiab] or "linum usitatissimum oil" [tiab] or "alpha-linolenic acid" [tiab]	RCT, Humans, English	1,737
Hypertension/Inflammation (O)	"hypertension" [tiab] or "hypertensives" [tiab] or "hypertense" [tiab] or "systolic blood pressure" [tiab] or "diastolic blood pressure" [tiab] or "elevated blood pressure" [tiab] or "high blood pressure" [tiab] or "blood pressure" [tiab] or "arterial pressure" [tiab] or "arterial hypertension" [tiab] or "vascular pressure" [tiab] or "vascular resistance" [tiab] or "inflammation" [tiab] or "inflammatory" [tiab] or "interleukin" [tiab] or "IL-6" [tiab] or "interleukin-6" [tiab] or "TNF-α" [tiab] or "tumor necrosis factor alpha" [tiab] or "CRP" [tiab] or "c-reactive protein" [tiab]	RCT, Humans, English	70,264
Combined searches	#1 and #2	RCT, Humans, English	485
**Database:**	**CINAHL**		
**Date of search**	**10/15/23**		
**Topic**	**Search words**	**Filters Applied**	**#Of Articles**
Olive oil/ Flaxseed oil (I)	SU "olive oil" OR SU "extra virgin olive oil" OR SU "virgin olive oil" OR SU "olive pomace oil" OR SU "refined olive oil" OR SU "pure olive oil" OR SU "oleic acid" OR SU "unrefined olive oil" OR SU "olive fruit oil" OR SU "olive extract" OR SU "light olive oil" OR SU "monounsaturated fatty acids" OR SU "olea europaea oil" OR SU	Boolean/Phrase, English, Human, RCT, Apply equivalent subjects, Apply related	882

	"flaxseed oil" OR SU "flax oil" OR SU "linseed oil" OR SU "flax extract" OR SU "linseed extract" OR SU "linum usitatissimum oil" OR SU "alpha-linolenic acid"	words	
Hypertension/Inflammation (O)	SU "hypertension" OR SU "hypertensives" OR SU "hypertense" OR SU "systolic blood pressure" OR SU "diastolic blood pressure" OR SU "elevated blood pressure" OR SU "high blood pressure" OR SU "blood pressure" OR SU "arterial pressure" OR SU "arterial hypertension" OR SU "vascular pressure" OR SU "vascular resistance" OR SU "inflammation" OR SU "Inflammatory" OR SU "interleukin" OR SU "IL-6" OR SU "interleukin-6" OR SU "tnf-α" OR SU "tumor necrosis factor alpha" OR SU "CRP" OR SU "c-reactive protein"	Boolean/Phrase, English, Human, RCT, Apply equivalent subjects, Apply related words	208,248
Combined searches	#1 and #2	Boolean/Phrase, English, Human, RCT, Apply equivalent subjects, Apply related words	135
**Database:**	**Web Of Science**		
**Date of search**	**10/15/23**		
**Topic**	**Search words**	**Filters Applied**	**#Of Articles**
Olive oil/ Flaxseed oil (I)	TI=["olive oil"] OR TI=["extra virgin olive oil"] OR TI=["virgin olive oil"] OR TI=["olive pomace oil"] OR TI=["refined olive oil"] OR TI=["pure olive oil"] OR TI=["oleic acid"] OR TI=["unrefined olive oil"] OR TI=["olive fruit oil"] OR TI=["olive extract"] OR TI=["light olive oil"] OR TI=["monounsaturated fatty acids"] OR TI=["olea europaea oil"] OR TI=["flaxseed oil"] OR TI=["flax oil"] OR TI=["linseed oil"] OR TI=["flax extract"] OR TI=["linseed extract"] OR TI=["linum usitatissimum oil"] OR TI=["alpha-linolenic acid"]	Doc type; Article, Lang; English	13,369
Hypertension/ Inflammation (O)	TI=["hypertension"] OR TI=["hypertensives"] OR TI=["hypertense"] OR TI=["systolic blood pressure"] OR TI=["diastolic blood pressure"] OR TI=["elevated blood pressure"] OR TI=["high blood pressure"] OR TI=["blood pressure"] OR TI=["arterial pressure"] OR TI=["arterial hypertension"] OR TI=["vascular pressure"] OR TI=["vascular resistance"] OR TI=["inflammation"] OR TI=["inflammatory"] OR TI=["interleukin"] OR TI=["IL-6"] OR TI=["interleukin-6"] OR TI=["TNF-α"] OR TI=["tumor necrosis factor alpha"] OR TI=["CRP"] OR TI=["c-reactive protein"]	Doc type; Article, Lang; English	455,372
Combined searches	#1 and #2	Doc type; Article, Lang; English	322
**Database:**	**Medline (Ovid)**		
**Date of search**	**10/16/23**		
**Topic**	**Search words**	**Filters Applied**	**#Of Articles**
Olive oil/Flaxseed oil (I)	"olive oil" or "extra virgin olive oil" or "virgin olive oil" or "olive pomace oil" or "refined olive oil" or "pure olive oil" or "oleic acid" or "unrefined olive oil" or "olive fruit oil" or "olive extract" or "light olive oil" or "monounsaturated fatty acids" or "olea europaea oil" or "flaxseed oil" or "flax oil" or "linseed oil" or "flax extract" or "linseed extract" or "linum usitatissimum oil" or "alpha-linolenic acid"	Title, Humans, English	8205
Hypertension/Inflammation (O)	"hypertension" or "hypertensives" or "hypertense" or "systolic blood pressure" or "diastolic blood pressure" or "elevated blood pressure" or "high blood pressure" or "blood pressure" or "arterial pressure" or "arterial hypertension" or "vascular pressure" or "vascular resistance" or "inflammation" or "inflammatory" or "interleukin" or "IL-6" or "interleukin-6" or "TNF-α" or "tumor necrosis factor alpha" or "CRP" or "c-reactive protein"	Title, Humans, English	661923
Combined searches	#1 and #2	Title, RCT, Humans, English	148

APPENDIX III. TITLE AND ABSTRACT SCREENING FORM.

Q1.) Is the article an original study?	Yes<br>No<br>If "no", submit form without proceeding further.<br>If unsure, submit to full text review	*Exclude:* Review articles, systematic reviews, scoping reviews, narrative reviews, systematic reviews and meta analysis
Q2.) Is the study peer-reviewed?	Yes<br>No<br>If "no", submit form without<br>Proceeding further.<br>If unsure, submit to full text<br>review.	*Exclude:* Study protocols, conference paper/abstracts, thesis, gray literature.
Q3.) Does the article involve human subjects?	Yes<br>No<br>If "no", submit form without<br>Proceeding further.<br>If unsure, submit to full text<br>review.	*Exclude:* Animals studies, in vitro studies
Q4.) Is the article a randomized controlled trial?	Yes<br>No<br>If "no", submit form without<br>Proceeding further.<br>If unsure, submit to full text<br>review.	*Exclude:* observational studies
Q5.) Is oral dietary supplementation involved in the study intervention?	Yes<br>No<br>If "no", submit form without<br>Proceeding further.<br>If unsure, submit to full text<br>review.	*Include:* High Polyphenol OO Intervention Low Polyphenol OO Intervention
Q6.) Does the intervention involve Olive Oil, Extra Virgin Olive Oil, Virgin Olive Oil, Olive Pomace Oil, Refined Olive Oil, Pure Olive Oil, Unrefined Olive Oil, Olive Fruit Oil, Olive Extract, Light Olive Oil, Olea Europaea Oil, Flaxseed Oil, Flax Oil, Linseed Oil, Flax Extract, Linseed Extract, Linum Usitatissimum Oil?	Yes<br>No<br>If "no", submit form without<br>Proceeding further.<br>If unsure, submit to full text<br>review.	-
Q7.) Is the placebo olive oil or flaxseed oil?	Yes<br>No<br>If "no", submit form without<br>Proceeding further.<br>If unsure, submit to full text<br>review.	-
Q6.) Is the intervention administered to adults?	Yes<br>No	Exclude: children, adolescents

APPENDIX IV. FULL TEXT SCREENING FORM.

Q1: Is the article written in English?	Yes<br>No	-
Q2: Is the article a randomized controlled trial?	Yes<br>No	-
Q3: Is the article a human trial?	Yes<br>No	-
Q4: Does the intervention include the mediterranean diet?	Yes<br>No	*Exclude*: Med diet supplemented with oils in any form
Q5: Is the intervention given *via* capsules?	Yes<br>No	-
Q6: Are subjects normotensive, prehypertensive, stage I hypertensive	Yes<br>No	*Exclude:* High risk stage 1 hypertensive, stage 2 hypertensive based on the 2017 AHA blood pressure categorization
Q7: Do the outcomes include blood pressure?	Yes<br>No	*Exclude:* Studies with no outcomes measuring blood pressure (SBP and/or DBP)<br>Include: Studies with blood pressure (SBPand/or DBP) and at least one inflammatory marker (CRP, TNF-a, IL-6)
Q8: Is the intervention less than 2 weeks?	Yes<br>No	-
Q9: Is olive oil the placebo or control?	Yes<br>No	*Include:* Olive oil as placebo

APPENDIX V. DATA EXTRACTION FORM.

Question	Options	Comments
Q1) What is the title of the paper?	Specify;	-
Q2.) Lead author	Specify;	-
Q3.) What is the country in which the study is conducted?	United States<br>UK<br>Canada<br>Australia<br>Other	If other, specify;
Q4.) Was the study conducted at multiple centers?	Yes<br>No<br>Unclear	-
Q5.) What is the study design?	Randomized controlled trial<br>Other	-
Q6.) How was the study randomized?	Specify;	-
Q7.) How were participants allocated to treatments?	Specify;	-
Q8.) What is the duration of the intervention?	Specify;	-
Q9.) What was the mode of delivery of the supplementation?	Beverage<br>Food/Meals<br>Pill/Capsules<br>Other, Specify:<br>Unclear	-

Q10.) What is the intervention dosage?	Specify;	-
Q11.) Was the olive oil the control?	Yes<br>No<br>If No, specify control:	-
Q12.) What is the oil grade?	Extra virgin olive oil<br>Virgin olive oil<br>Refined olive oil<br>Olive oil pomace<br>Refined flaxseed oil<br>Not specified	-
Q13.) Was the intervention/control groups measured? If yes, how?	Yes, Specify<br>No<br>Unclear	-
Q14.) Diet recommendation?	Yes, Specify<br>No<br>Not, Specified	-
Q15.) Was exercise controlled? If so, how?	Yes, Specify<br>No<br>Unclear	--
Q16.) What was the race/ethnicity of participants?	Specify;	--
Q17.) What was the age of the participants?	Specify;	--
Q18.) What was the sex of the participants?	Male only<br>Female only<br>Both	--
Q19.) What disease state are participants at risk for?	Specify;	-
Q20.) What setting was the study intervention administered?	Specify;	-
Q21.) What is the inclusion criteria?	Specify;	-
Q22.) What is the exclusion criteria?	Specify;	-
Q23.) Method of recruitment of participants	Phone<br>Mail<br>Clinic patients<br>Voluntary<br>Other, Specify;	-
Q24.) What is the total number of participants & number of participants in intervention group?	Specify;	-
Q25.) Blood pressure categorization?	Normotension<br>Prehypertension<br>Stage I Hypertension	-
Q26.) What outcomes were measured?	Blood pressure<br>CRP<br>TNFα<br>IL-6	-
Q27.) Describe the significance of any outcomes measured.	Specify;	-
Q28.) What is the study funding sources?	Specify;	-
Q29.) Are any outcomes of significance reported for a possible meta-analysis?	Specify;	-
